# Supplementary material for: Atomic scale crystal field mapping of polar vortices in oxide superlattices
Source: Nat Commun. 2021 Nov 1;12:6273. doi: 10.1038/s41467-021-26476-5 (PMC8560910; doi:10.1038/s41467-021-26476-5)
Supplement: Supplementary file 1 — Supplementary Information [file 41467_2021_26476_MOESM1_ESM.pdf]

Supplemental Material for:
Atomic scale crystal field mapping of the polar vortices in oxide superlattices.

Sandhya Susarla[1,2,3*], Pablo García-Fernández[4], Colin Ophus[1], Sujit Das[2], Pablo Aguado-Puente[5], Margaret McCarter[2,3], Peter Ercius[1],Lane W. Martin[2,3], Ramamoorthy Ramesh[1,2,3,6*], Javier Junquera[4*]

1. National Center for Electron Microscopy, Molecular Foundry, Lawrence Berkeley National Laboratory, Berkeley, California, 94720, USA
2. Department of Materials Science and Engineering, University of California, Berkeley, California, 94720, USA
3. Materials Sciences Division, Lawrence Berkeley National Laboratory, Berkeley, California, 94720, USA
4. Departamento de Ciencias de la Tierra y Física de la Materia Condensada, Universidad de Cantabria, Cantabria Campus Internacional, Avenida de los Castros s/n, 39005 Santander, Spain
5. Atomistic Simulation Centre, Queen's University Belfast, Belfast BT7 1NN, UK
6. Department of Physics, University of California, Berkeley, CA 94720, USA

*Corresponding author
Email: ssusarla@lbl.gov , rramesh@berkeley.edu , javier.junquera@unican.es

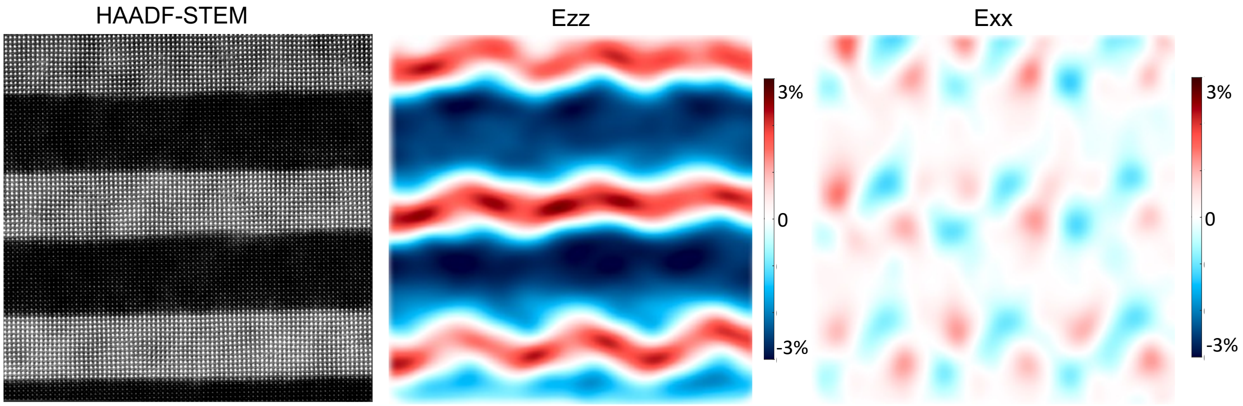

Supplementary Figure 1: HAADF STEM image of PTO/STO superlattices with the corresponding out of plane and in-plane strain maps respectively.

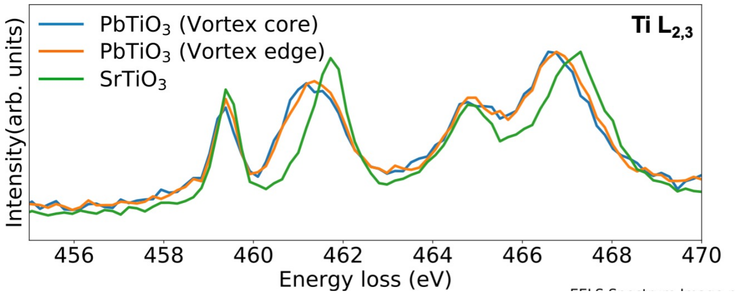

Supplementary Figure 2: Average raw data of $PbTiO_3$ (vortex core), $PbTiO_3$ (vortex edge), $SrTiO_3$ extracted from 1350 pixels (summing six 15x15 multiple equivalent areas indicated in Fig. 2(a) of the main body of the manuscript).

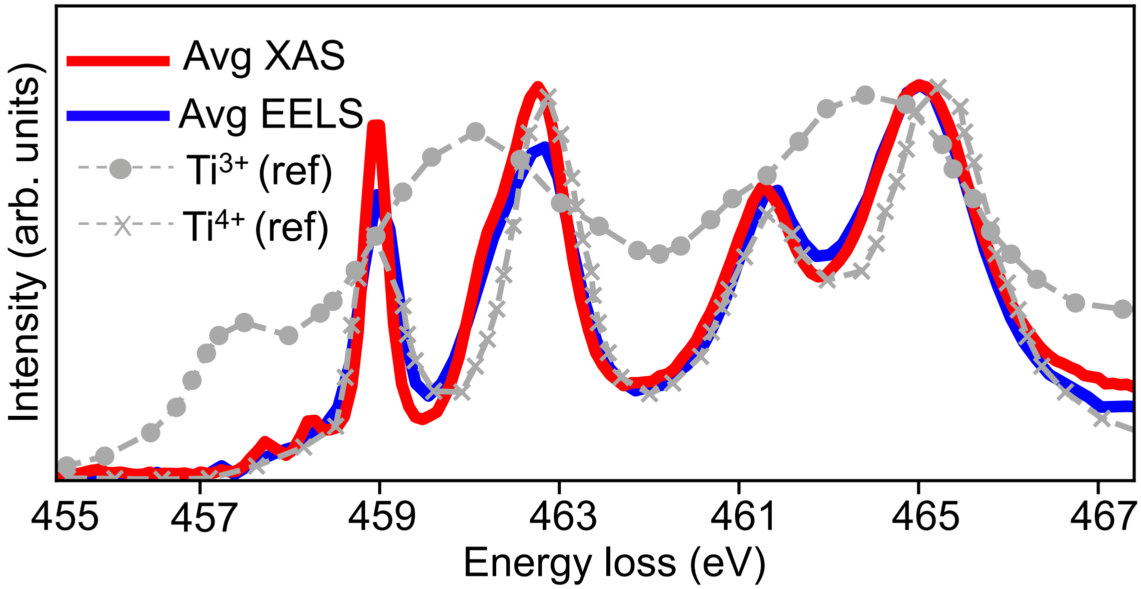

Supplementary Figure 3. Average XAS and EELS spectra in comparison to Ti^{3+} and Ti^{4+} from literature.[1,2]

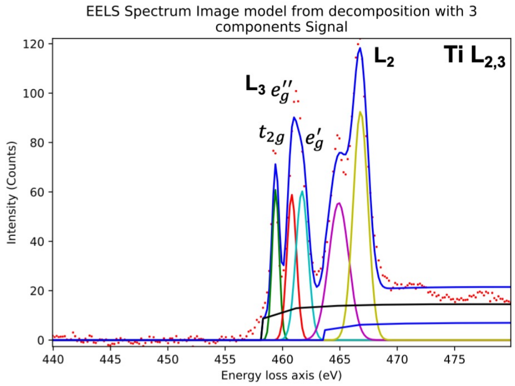

Supplementary Figure 4. The Gaussian fitted spectra from a randomly selected single pixel in EELS map in
Fig 3.

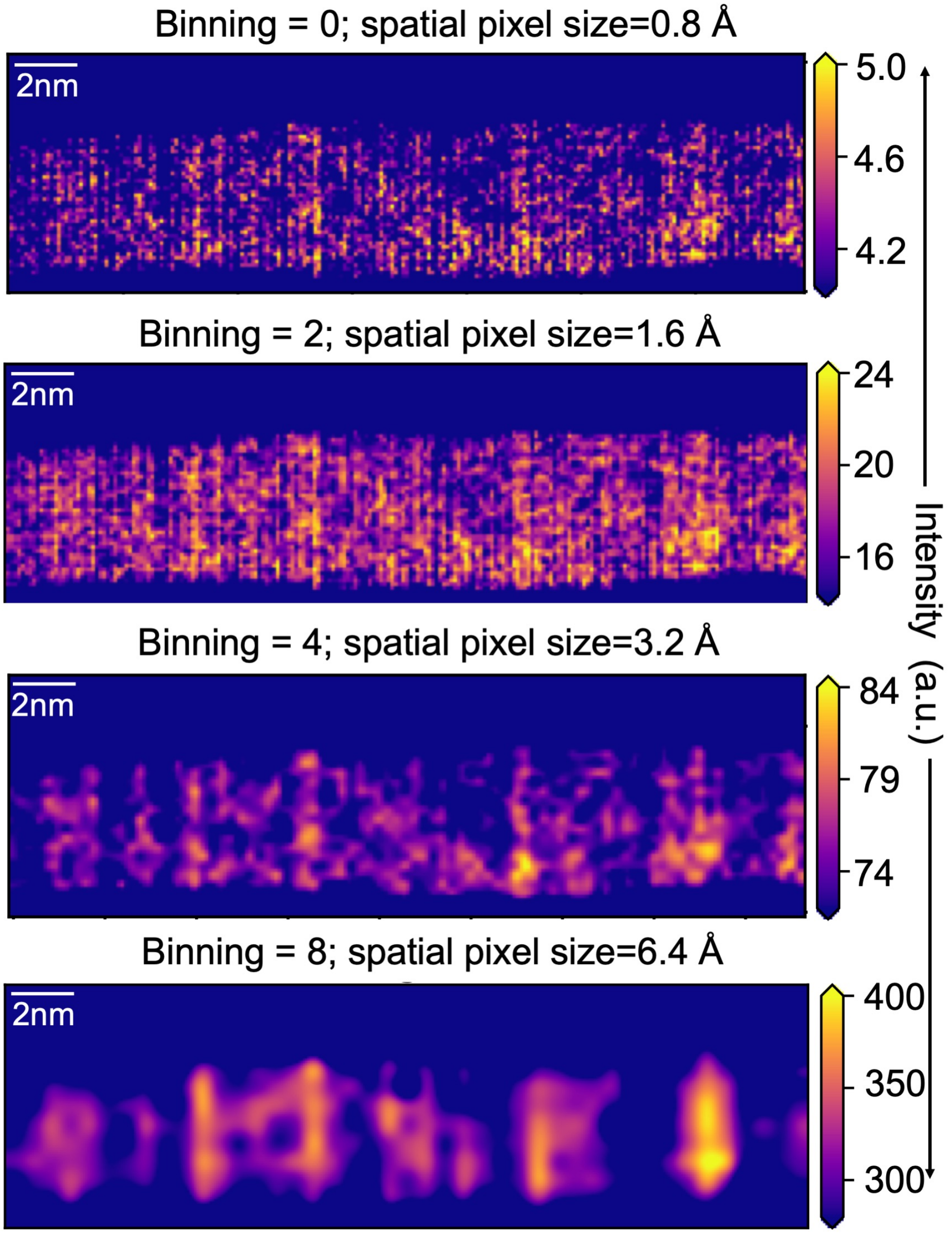

Supplementary Figure 5: The intensity of the e_g'' peak extracted by Gaussian fitting from STEM-EELS data with 0, 2,4 and 8 binning values applied in real space.

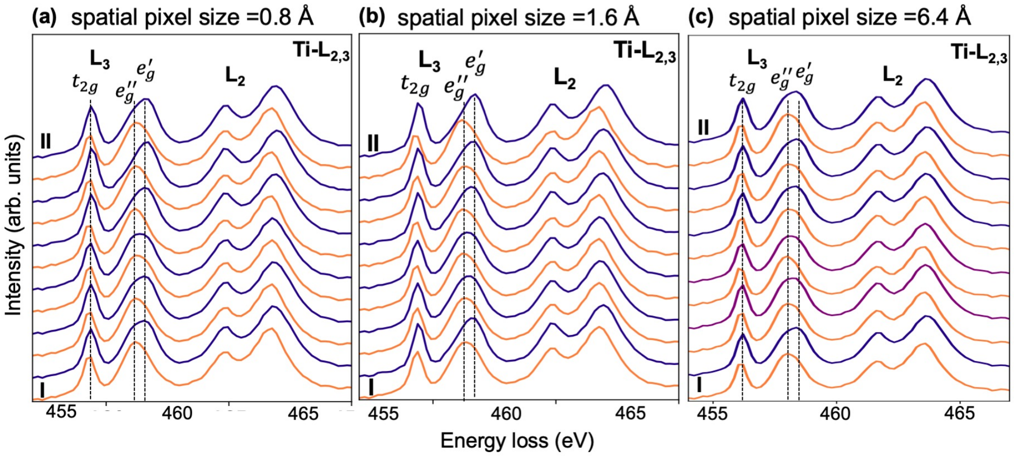

Supplementary Figure 6. Comparison of single spectra with spatial pixel sizes of a) 0.8 Å b) 1.6 Å and c) 6.4 Å extracted from the regions indicated in Fig. 3a. Note that the spectra do not change with the pixel size changing from 0.8 Å to 6.4 Å.

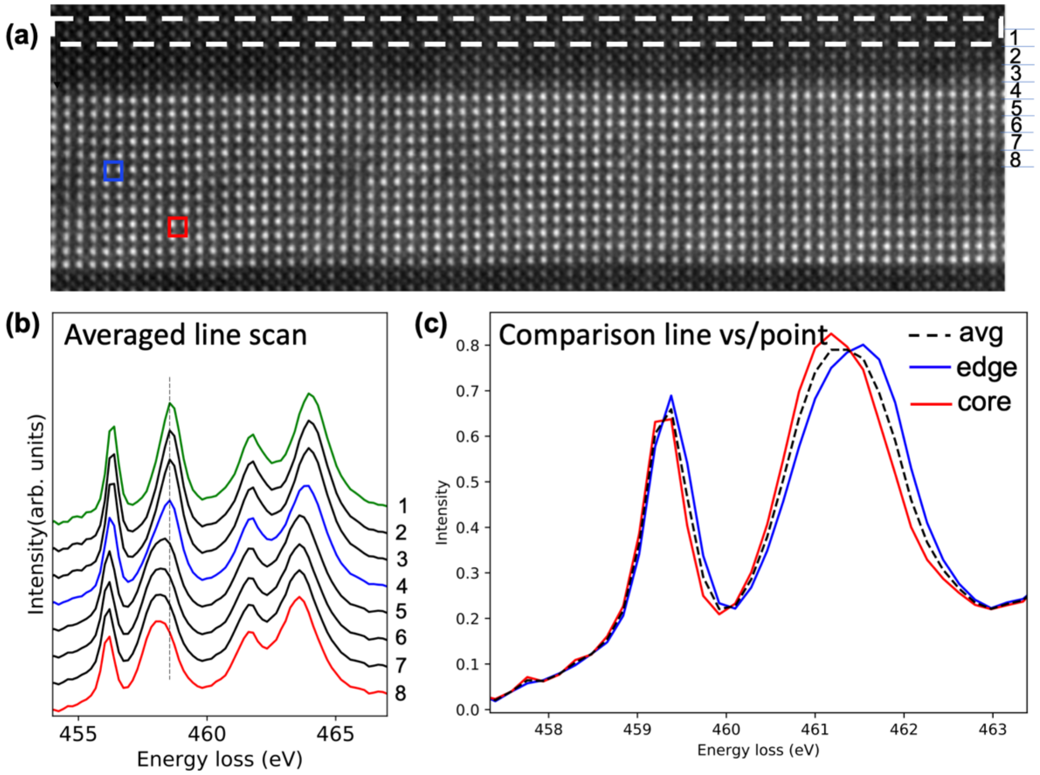

Supplementary Figure 7. (a) HAADF-STEM image displaying PTO/STO superlattices. (b) EEL spectra from row 1 to row 8 as indicated in panel (a). (c) Comparison of point spectrum shown in the main draft to line spectrum indicated in (b).

Note 1: Layer by layer projected density of states (PDOS)

First-principles calculations were carried out in a $(PbTiO_3)_6/(SrTiO_3)_6$ superlattice, mostly due to computational efficiency. The simulation box required to include the vortices in a 6/6 superlattice already amounts to 720 atoms resulting in a long computation time. Changing to a 16/16 superlattice would make the calculations prohibitively computationally expensive. Further, the 6/6 calculations can be accurately extrapolated to interpret the experiments with the 16/16 periodicity because from the structural point of view, the structure we are analyzing in this work from first principles in 6/6 superlattices is essentially the same as the one obtained from second-principles simulations in 14/14 superlattices, where we can complete relaxations of much larger supercells. As shown in the Supplementary Figure 8, the presence of ordered arrays of clockwise/counterclockwise vortices within the $PbTiO_3$ layer is clearly reproduced in both cases.

Unfortunately, the current version of our second-principles code does not allow the computation of the band structures, the basic ingredient to analyze the EELS spectra.

In Supplementary Figure 9 we show the layer by layer spatially resolved projected density of states (PDOS) on the atomic *d*-orbitals of the Ti atoms. A line cut along a vortex edge with the polarization pointing up has been selected.

The center of mass of these PDOS are used to estimate the energy levels of the different orbitals in order to compute the CFS, Δe_g, and Δt_{2g} splittings. Similar projections are taken for other Ti at different places of the vortex, as indicated in **Fig. 4(a)** of the main body of the manuscript.

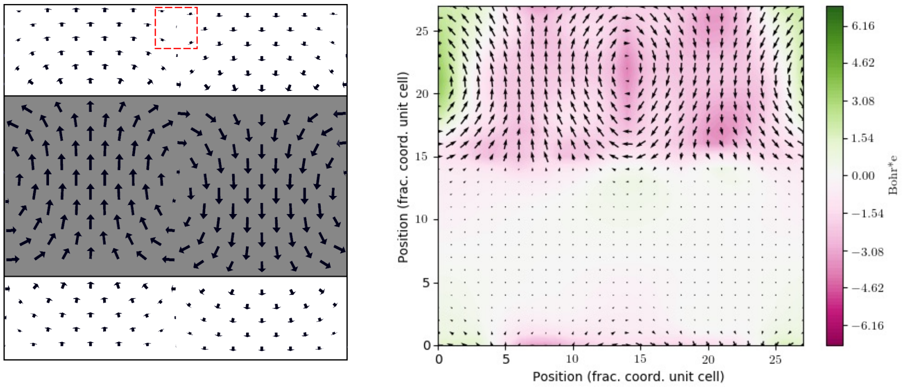

Supplementary Figure 8. Simulations on the same $PbTiO_3/SrTiO_3$ superlattice system using different methods. Left: first-principles simulations in a 6/6 superlattice. Right: second-principles method using a 14/14 superlattice.

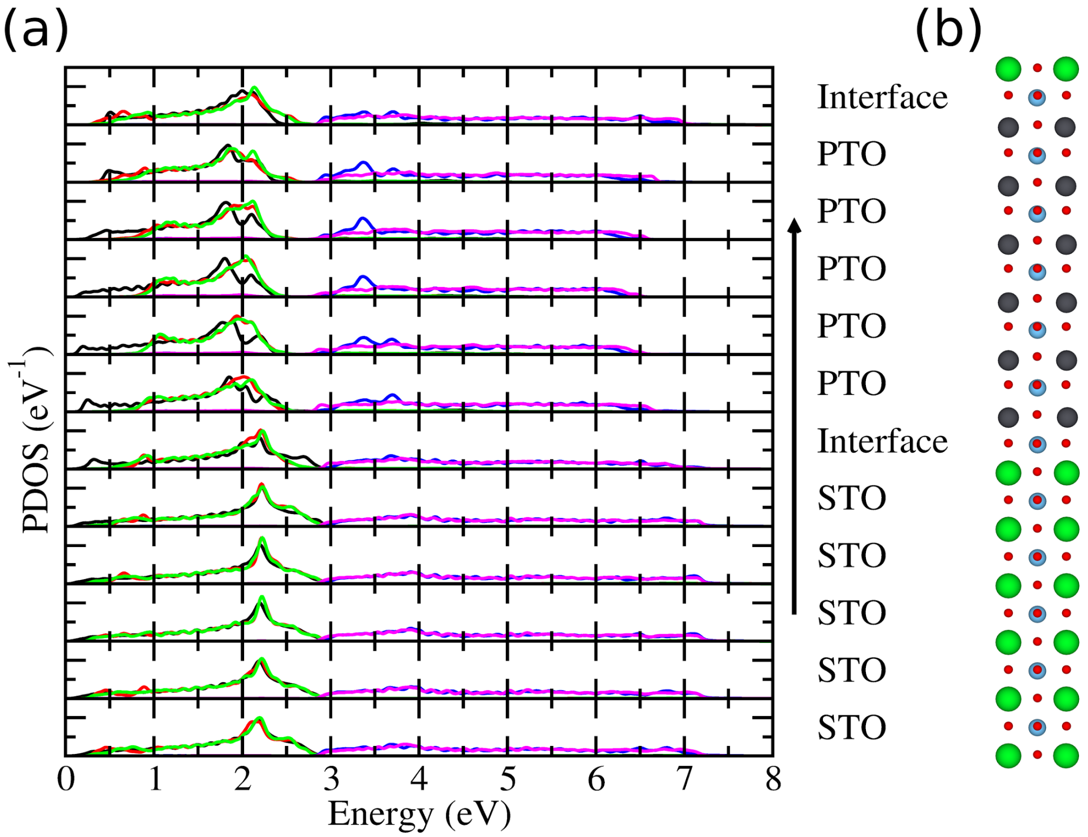

Supplementary Figure 9. (a) Layer-by-layer PDOS on the atomic *d*-orbitals of the Ti atoms for the corresponding layer at the same height as in panel (b). Solid black lines represent the projection on the d_{xy} orbitals, red on d_{yz}, green on d_{xz}, blue on d_{3z2-r2}, and magenta on d_{x2-y2}. (b) Schematic representation of one column of $(PbTiO_3)_6/(SrTiO_3)_6$ superlattice at the vortex edge (i.e. at the center of a domain) with polarization up. Sr atoms are represented by green balls, Pb by grey, Ti by blue and O by red spheres. Zero of energies is taken at the bottom of the conduction band.

Note 2: Bulk DFT calculations and evolution of spectroscopic parameters with the distortion of the cubic perovskite cell

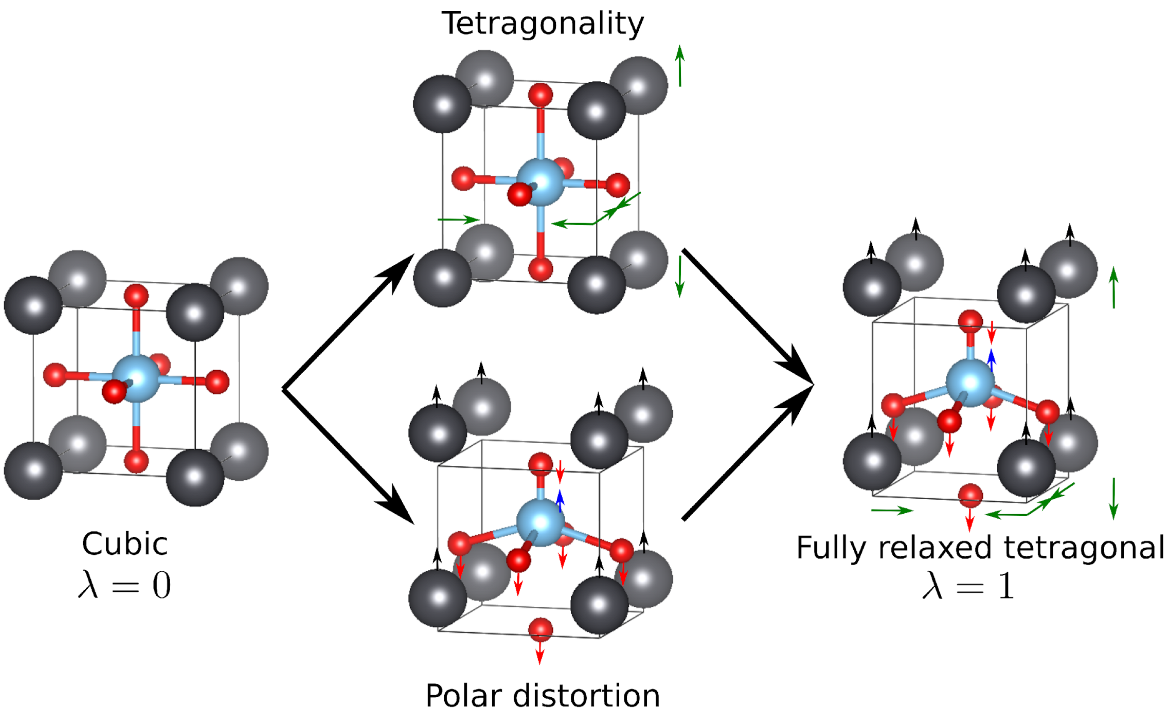

Supplementary Figure 10. The distortions considered to study the variation of the spectroscopic parameters. On the left, for λ=0, we have the relaxed cubic structure and, on the right, for λ=1, the relaxed tetragonal structure. In the middle we show the two distortions that, combined, lead to the final tetragonal structure. At the central top panel, the cartoon represents the tetragonality that only affects the cell shape (green arrows), but not the fractional coordinates of the atoms. At the central bottom panel, the cartoon represents the polar distortion that only affects the atomic positions (in red, blue, and black for oxygen, titanium, and lead, respectively).

In order to understand the effects of local changes on the EELS spectra inside the PTO/STO superlattices and reflected in the spectroscopic parameters Δe_g, Δt_{2g} and CFS, we have carried out simulations in bulk PTO and STO for different geometries described in Supplementary Figure 10. We consider as a reference configuration the cubic structure of each of these systems that are characterized in our LDA simulations by a lattice parameter of 3.89 Å and 3.87 Å, respectively. When a full geometry relaxation is carried out, it is found that PTO undergoes a symmetry breaking distortion that leads to the characteristic polar tetragonal structure of this ferroelectric material.

Two main distortions determine this structure; first the polar mode associated to the displacement of the ions inside the unit cell of PTO and, second, the concomitant cell shape change from cubic to elongated tetragonal, usually accounted for through a tetragonality ratio *c/a* >1.0. These distortions are illustrated in Supplementary Figure 10.

In order to go smoothly from the cubic ($\mathbf{R}_{\text{cubic}}$) to the fully relaxed tetragonal structure ($\mathbf{R}_{\text{tetra}}$) we define the linear transformation:

$$\mathbf{R}(\lambda)= \mathbf{R}_{\text{cubic}}+\lambda(\mathbf{R}_{\text{tetra}}-\mathbf{R}_{\text{cubic}}) \quad (1)$$

where $\mathbf{R}(\lambda)$ is the studied geometry and $\mathbf{R}_{\text{cubic}}$ and $\mathbf{R}_{\text{tetra}}$ are, respectively, the relaxed PTO cubic and tetragonal geometries. Thus, for $\lambda=0$ we obtain the cubic structure and for $\lambda=1$ a tetragonal structure.

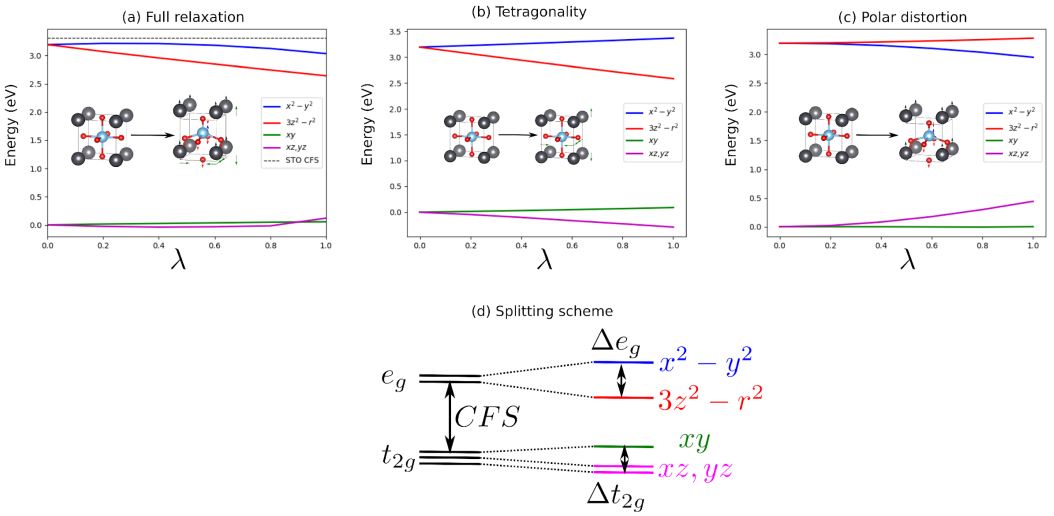

Supplementary Figure 11. The center-of-mass for the mainly $3z^2$-r^2, x^2-y^2, *xy*, *xz* and *yz* bands following the cubic to tetragonal distortion. In (a) all the energy levels are evaluated for the full distortion. The dashed line represents the CFS of the bulk STO cubic lattice. In (b) only the cell is distorted to measure the effect of tetragonality. In (c) only the atomic positions are changed, in order to evaluate the effect of the polar distortion. Finally, in (d) a qualitative scheme with the illustration of the various spectroscopic parameters (CFS, Δt_{2g}, Δe_g) for the cubic (left) and tetragonal (right) phases is shown. The zero of energies is taken at the center of mass of the projected density of states for the *xy* orbital.

Using this transformation we can plot the center-of-mass of the projected density of states on the Ti 3*d* bands forming the low-energy region of the conduction band. In particular, we employ the projected density of states for *xy*, *xz*, *yz*, $3z^2$-r^2 and x^2-y^2 orbitals. As can be seen in Supplementary Figure 11(a), and as expected for cubic geometry, for λ=0 the first three levels (*xy*, *xz* and *yz*) condense into a triply degenerate t_{2g} state (Δt_{2g}=0) while $3z^2$-r^2 and x^2-y^2 are also degenerate forming an e_g level (Δe_g=0). The separation between these two states, the e_g-t_{2g} splitting, is what we have called the crystal-field-splitting (CFS). The CFS in PTO is consistently smaller than that of cubic STO (shown as a dashed black line in Supplementary Figure 11(a)). This is in agreement with the well-known fact [3] that the CFS is very sensitive to the average metal-ligand distance, increasing as the Ti-O bond shortens. Given that the Ti-O distance in cubic PTO is larger than the corresponding distance in STO, the CFS is larger in the latter lattice than in the former.

As the distortion is activated and increases (λ>0) we observe that both the t_{2g} and e_g levels split, leading to non-null Δe_g and Δt_{2g} values. The t_{2g} level splits into a non-degenerate *xy* (b_2 symmetry) and degenerate *xz*, *yz* (*e* symmetry) sublevels. The e_g breaks into $3z^2$-r^2 (a_1 symmetry) and x^2-y^2 (b_1 symmetry) components, a behavior consistent with going from O_h to C_{4v} symmetry (see Supplementary Figure 11(d)). The observed splitting in the t_{2g} level is quite small, particularly when compared to that of taking place in the e_g states. As can be observed the energy of the x^2-y^2 state stays above the $3z^2$-r^2 orbital leading to a positive Δe_g splitting that increases with the distortion.

In order to study the origin of these splittings we will now proceed to disentangle the effect of each of the main distortions on these parameters. In order to do so we will plot the center-of-

mass of the bands when (i) only the distortion of the cell is considered, while leaving the atoms at the fractional coordinates characteristic of the cubic phase and (ii) changing the fractional position of the atoms, leaving the cubic phase cell unchanged. In this way we can study how the tetragonality (i) and polarization (ii) alter, individually, the energy levels. These results are plotted in Supplementary Figure 11(b) and (c), respectively.

When we study the effect of tetragonality (Supplementary Figure 11 (b)) we see that the in-plane orbitals (x^2-y^2 and xy) linearly increase their energy, while those with an out-of-plane component ($3z^2$-r^2 and (xz,yz)) decrease it. This result can be understood looking at the Ti-O distances, particularly comparing what happens to them in the plane perpendicular to the tetragonal axis and in the direction of this axis. In the plane, the Ti-O distance decreases with λ leading to an increase of the energy of (antibonding) orbitals localized in the plane, i.e. x^2-y^2 and xy. In the axis, the increase of c/a makes the Ti-O distances longer, reducing the energy of orbitals oriented in this direction ($3z^2$-r^2 and (xz,yz)). These energy variations lead to positive Δe_g and Δt_{2g} splitting when λ is positive (i.e., when $c/a > 1.0$) and would lead to negative splitting if λ became negative (i. e., when $c/a < 1.0$). The effect is significantly stronger on σ-antibonding levels (e_g) than in π-antibonding ones (t_{2g}) given that the distortion symmetrically alters distances but not bond angles.

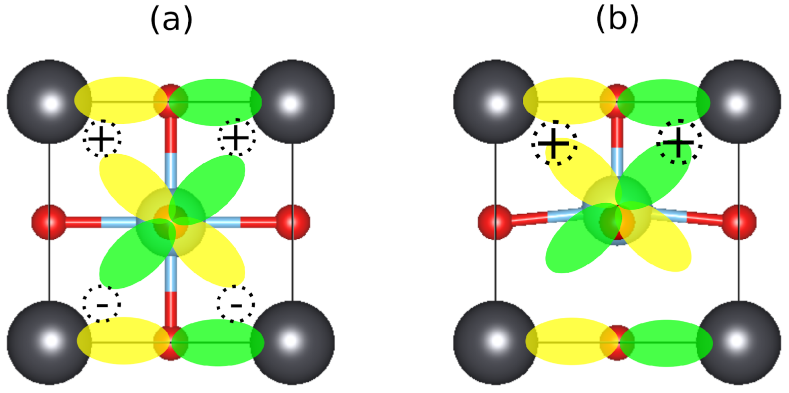

Supplementary Figure S12. Change in the overlap between the titanium d_{xz} orbital and oxygen p_π upon the polar distortion on the (initially) non-bonding orbital in the cubic TiO_6 complex. (a) Occupied non-bonding, mainly oxygen p_π orbital of the cubic TiO_6 complex. Note that the overlap

of the d_{xz} with top and bottom oxygen orbitals is perfectly compensated (b) The same orbital when Ti moves off-center. In this case the overlap with top and bottom orbitals is not compensated leading to an overall bonding orbital

When we study the effect of the polar distortion (Supplementary Figure 11 (c)) we see that, in this case, in-plane orbitals (x^2-y^2 and xy) quadratically decrease their energy while those with an out-of-plane component ($3z^2$-r^2 and (xz,yz)) increase it, in contrast with the effect of the tetragonality. In this case, the effect on the t_{2g} splitting is somewhat larger than in the e_g orbitals and is associated to the asymmetric changes of the overlap between metal (xz,yz) orbitals with oxygen p_π orbitals when the bond angle changes (see Supplementary Figure 12). It is important to realize that while the tetragonal distortion leads to $\Delta e_g>0$ and $\Delta t_{2g}>0$ the opposite happens with the polar distortion. In this case, and given that the behavior is quadratic the splitting would be the same for positive or negative λ.

Going back to Supplementary Figure 11(a) we see that, when adding the effects of both distortions, the dominant effect in Δe_g is the tetragonality leading to $\Delta e_g>0$. However, it is important to note that a significant change in the tetragonality (lowering the *c/a* ratio) that retains most of the polarization may make the latter effect dominant leading to $\Delta e_g<0$ (as it occurs for the Ti-3 close to the core of the vortex in **Fig. 4** of the main body of the manuscript) or when the polarization becomes small (as it occurs close to the interfaces; Ti-1, **Fig. 4** of the main body of the manuscript and vortex core (Ti-5)) it becomes large. However in the case of Δt_{2g}, for all λ values the polarization and tetragonality compensate each other, leading to a small splitting for all values of λ. However, this splitting can become significant when the tetragonality and polar distortion are varied individually keeping the other constant.

In PTO/STO superlattices a third effect becomes important which is the rotation of the polarization. Performing the same kind of analysis as above is not possible as it is important to

project the density of states on orbitals with the correct quantization axis. Thus, in the previous tetragonal situations the z axis was the main symmetry axis and projection was carried out on the $3z^2$-r^2, x^2-y^2, xy, xz and yz orbitals. However, if the polarization is observed above or below the vortex core (see **Fig. 4** of the main body of the manuscript) we can see that the polarization points in the x direction and the correct orbitals to project the density of states would be $3x^2$-r^2, z^2-y^2, zy, xz and yz. In order to avoid problems with the quantization axis we analyze the initial situation (polarization pointing in the direction of the positive z axis) and the final one (polarization pointing in the direction of the positive x axis) without discussing intermediate geometries. The first situation (polarization along z) can be simply represented by the previous calculations when λ=1. Moreover, in bulk, when the polarization points along the x-axis, the situation is completely equivalent to the case when it points along z. However, in DSO-grown PTO/STO superlattices the in-plane lattice constants (a,b) are fixed by the substrate. Thus, the lattice constant in x and y is fixed to that of DSO while the one along z can be accommodated to minimize the energy of the system. That means that when the polarization runs along z the equatorial (with respect to the polarization) lattice constants are fixed while the longitudinal one is free to change, while in the case when it runs along x the lattice constants that are fixed are the longitudinal and one equatorial, while the second equatorial is free to change (see Supplementary Figure 13(a)). If we now take the λ=1 case as a reference and we increase an equatorial lattice constant to simulate what is happening above the vortex core we can see that the x^2-y^2 orbital energy decays sharply leading to a negative contribution to Δe_g as shown in Supplementary Figure 13(b).

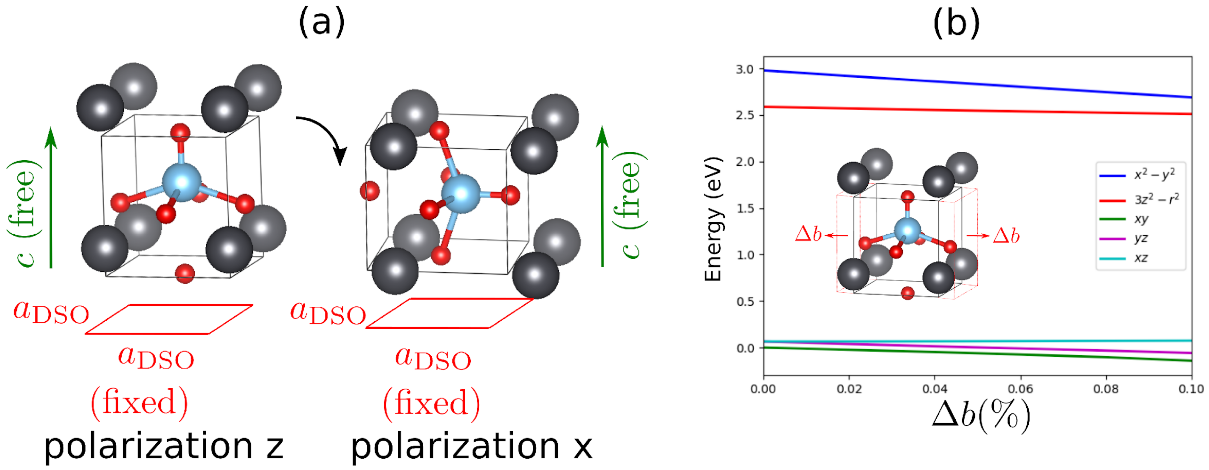

Supplementary Figure S13. (a) Illustration of the initial and final geometries as the polarization rotates from the z to the x axis. In red we show the constraints imposed by the substrate to the lattice constant the direction along which the lattice constant is free to change is shown in green. As the system rotates the polarization from the z-axis to the x-axis it goes from tetragonal (z-axis) to orthorhombic (x-axis) due to the constraints imposed on the lattice constants by the substrate. (b) Variation of the band center-of-mass as the orthorhombicity of the system is changed (the lattice constant b is increased keeping a and c fixed).

References:

1. Ohtomo, A., Muller, D. A., Grazul, J. L. & Hwang, H. Y. Artificial charge-modulationin atomic-scale perovskite titanate superlattices. *Nature* **419**, 378–380 (2002).
2. Cao, Y. *et al.* Engineered Mott ground state in a LaTiO(3+δ)/LaNiO3 heterostructure. *Nat. Commun.* **7**, 10418 (2016).
3. Williams, A. F. Electronic Spectra and Magnetic Properties of Inorganic Compounds. in *A Theoretical Approach to Inorganic Chemistry* (ed. Williams, A. F.) 132–158 (Springer Berlin Heidelberg, 1979).
